# Impact of national health city campaign on public health in China

**DOI:** 10.3389/fpubh.2025.1594104

**Published:** 2025-05-01

**Authors:** Wenjing Wang, Songjiang Liu, Bingnan Guo

**Affiliations:** ^1^School of Humanities and Social Sciences, Jiangsu University of Science and Technology, Zhenjiang, China; ^2^School of Economics and Management, Gannan University of Science and Technology, Ganzhou, China

**Keywords:** national health city, public health level, DID model, PSM-DID model, urban environmental governance

## Abstract

**Introduction:**

Establishing a national health city is an important national policy aimed at optimizing urban environment and improving residents’ health. A rigorous evaluation of the policy’s impact on China’s public health is imperative. Such an evaluation would provide a scientific foundation for the continuous improvement of the policy and enhance the overall health of the population.

**Methods:**

Based on the panel data of 84 prefecture-level cities in China from 2011 to 2023, this study takes healthy cities as a quasi-natural experiment, and employs a DID model to deeply explore the impact of the National Health City Campaign (NHCC) on public health in China.

**Results:**

The research indicates that the policy has led to a decline in mortality rates and has contributed to the enhancement of public health in China to a certain extent. The impact of the NHCC varies across different regions, economic development levels, and population sizes. In addition, this study determined that the NHCC is capable of achieving substantial enhancements in public health levels by promoting the optimization and upgrading of industrial structure. The impact of the sewage treatment rate on public health levels is found to be regulatory in nature.

**Discussion:**

This study is instrumental in enhancing public health policy, providing a theoretical foundation for the development of a healthy city, and plays a pivotal role in the promotion of the Healthy China Initiative.

## 1 Introduction

Human health is confronting a spectrum of emerging risks and challenges, driven by the concurrent advancement of industrialization, urbanization, and population aging, alongside evolving societal aspirations for enhanced living standards. In 1989, the World Health Organization (WHO) first put forward the concept of healthy city. As an important part of sustainable urban development, the Healthy Cities Movement aims to improve the well-being of urban populations through strategic planning and decision-making. This approach includes incorporating health considerations into urban planning and development processes, with emphasis on creating an environment and social welfare that promotes physical, mental and psychological health (1). With the formal proposal of healthy cities, the movement of healthy cities has been launched all over the world. The global assessment shows the differences in the level of healthy development among countries in the world, among which cities such as Singapore and Amsterdam have performed well, while cities such as Dubai and Johannesburg have lagged behind (2, 3).

As the core policy carrier for the construction of healthy cities in China, the “National Health City Campaign” (NHCC) policy has been an important practical innovation in China’s urban public health governance system since its launch in 1994. Through the “National Health City Standards,” this policy has established a scientific and rigorous evaluation index system. Its evaluation dimensions cover 10 major fields and 52 specific indicators, including urban appearance and environmental governance, disease prevention and control, and municipal facility construction, forming a three-dimensional governance framework that encompasses the urban environment, facilities, and services. From the perspective of the policy design mechanism, the NHCC takes environmental comprehensive governance as the foundation, the improvement of public health service capabilities as the core, and infrastructure construction as the support, focusing on constructing an urban development model that coordinates the prevention and control of health risks and the creation of a livable space.

From the perspective of international comparison, this policy has significant isomorphism with the WHO Healthy City Project in terms of value orientation. Strategically, both aim to enhance residents’ health and well-being by reducing the environmental health risk coefficient. However, at the policy implementation level, the NHCC has blazed an innovative trail with distinct Chinese features. In contrast to Singapore’s elaborate environmental management system established via the “Garden City” program or the low-carbon and healthy model advanced by Amsterdam under the “Circular Economy 2020–2025″ strategy, China’s NHCC policy has erected a three-dimensional governance network featuring government-led, enterprise-coordinated, and community-co-governed. This governance mechanism, with administrative impetus at its core, efficiently amalgamates cross-departmental resources by incorporating the establishment of healthy cities into the local government performance appraisal system. Amidst the rapid urbanization, it has orchestrated a focused push for public health infrastructure construction. Taking Suzhou as an illustrative case, it has integrated and innovated the National Healthy City Standards with the WHO’s healthy city indicators. By setting up a three-dimensional linkage mechanism that combines the execution of environmental governance plans, the drive for industrial upgrading and the development of community healthcare systems, following the policy implementation, the hospitalization rate for respiratory diseases has dropped by 22.3%. Consequently, Suzhou has become the world’s first city to be admitted into the WHO’s Healthy City Cooperation Network.

When the needs of society shift from economic development to healthy living, the concern for improving public health becomes an important yardstick for measuring public well-being. The core of public health is to realize the improvement of the health of all people and promote the sustainable development of society. However, high ecological pollution, medium and low technology industries (4–6), and unequal allocation of healthcare resources with uneven economic development (7) remain important threats.

**Figure 1:**
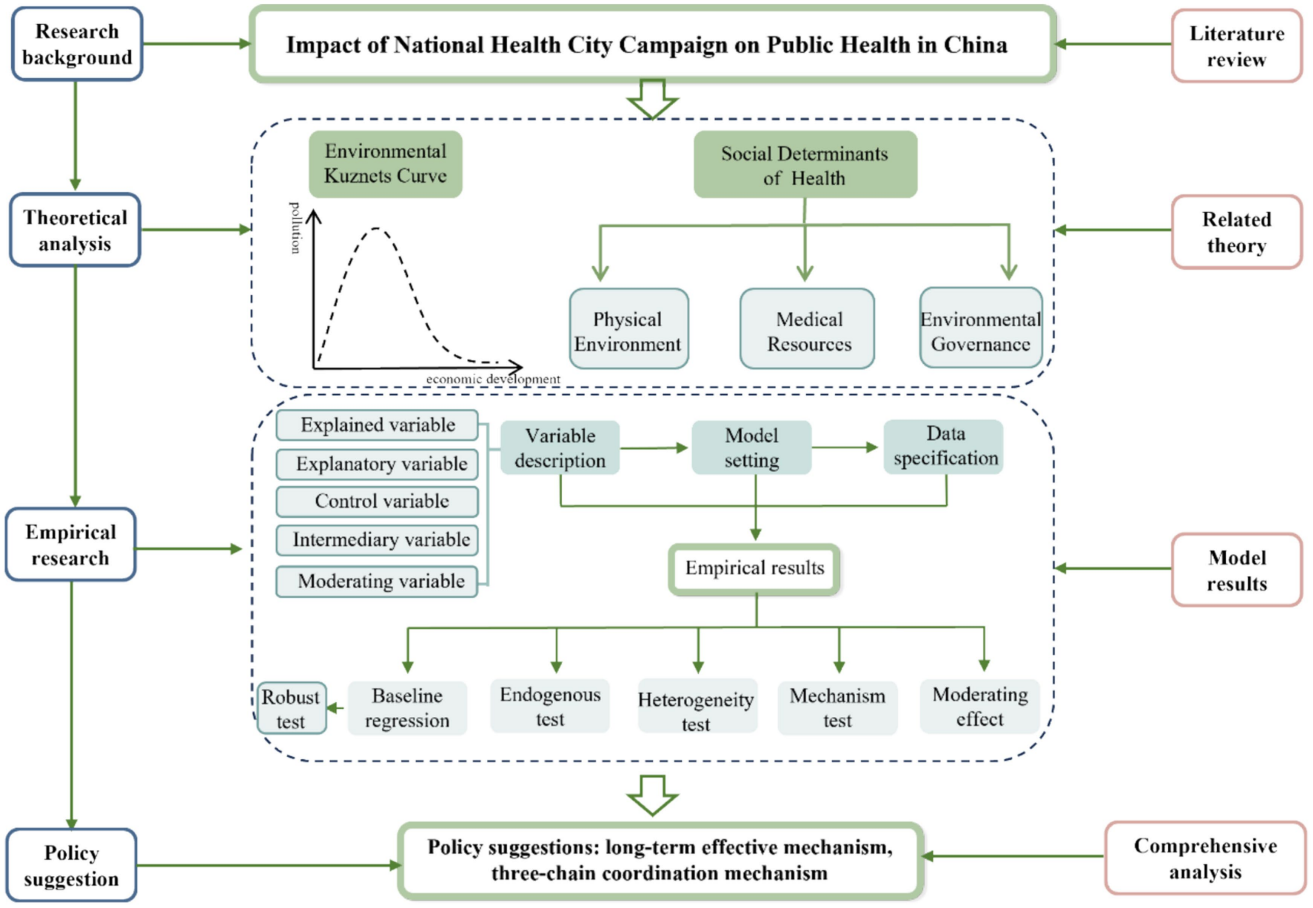
Framework structure diagram.

In the existing research, both medical research and economic research have affirmed the positive impact of the NHCC. So, will the NHCC improve public health in China? What are the mechanisms of impact? How does it vary across regions? In view of this, this study empirically analyzes whether the NHCC can reduce public health risks by using a Difference-in-Differences (DID) model and the Propensity Score Matching Difference-in-Differences (PSM-DID) Model. This study provides the basis and recommendations for the promotion of the Healthy China Initiative and the enhancement of China’s public well-being index. The framework structure is given in Figure 1.

## 2 Literature review

The literature closely related to this study is mainly divided into two branches: one is the research on healthy cities. The other is the research on the impact of healthy cities on public health.

The existing research on the NHCC mainly focuses on its origin, supporting measures and future efforts. The Healthy City Initiative has its origins in urban planning to improve public health. The Hygienic City Campaign of the 1840s and the Garden City Movement spanning the late 19th to early 20th century established the foundational framework for integrating health considerations into urban planning. In 1946, WHO defined the meaning of “health” for the first time, that is, physical, psychological and social well-being, which attracted great attention from all countries in the world. In 1984, in response to the health problems caused by rapid urbanization, WHO first put forward the concept of “healthy city,” which became an important force to promote this movement and promoted the healthy development of urban environment through comprehensive and cross-sectoral methods (8, 9). At present, research in academic circles on policies related to healthy cities focuses mainly on the following three aspects: First, health should be mainstreamed into all policies. The inclusion of health objectives in all areas of urban policy has been advocated to reduce health inequalities and increase social well-being (10). Effective policy implementation necessitates collaboration between city authorities, non-governmental organizations, private companies, and community groups (11). This kind of coordinated action is very important to address the complex factors that affect urban health. Third, comprehensive urban planning. The Healthy City Initiative emphasizes the integration of health considerations into the urban planning process, including ensuring that residents have access to clean water, sanitation facilities, green space and safe housing, while advocating active modes of transportation such as walking and cycling (9, 12). Although many cities around the world have adopted the framework of healthy cities, it is still necessary to systematically evaluate these initiatives to understand their effectiveness and to guide future efforts. Therefore, it is particularly important to formulate a sound evaluation plan for evaluating progress and identifying improvements (8, 13). In addition, there are significant differences in the development of healthy cities in different regions. In countries such as China, the imbalance in healthy urban development is more pronounced. Resolving these differences requires targeted efforts to promote the fairness of resource allocation and strengthen health-oriented urban planning (14, 15).

On the other hand, given that urban planning plays a key role in shaping the health of urban residents, academic circles have accumulated some achievements on the impact of healthy cities on public health. Effective urban planning can reduce the infection risk of non-communicable diseases by promoting physical exercise, improving air quality and enhancing social interaction through the planning of public spaces and transportation systems (16). The integration of health considerations into urban planning has been identified as a critical factor in creating environments that support healthy lifestyles and mitigate health disparities (8). Current research underscores three key strategies to enhance the public health impact of healthy city initiatives. First, the “health in all policies” approach, which mandates health integration across all governance domains, has gained renewed emphasis through innovations like the “15-min city” model. This urban design paradigm prioritizes equitable access to essential services within a 15-min walking or cycling radius, thereby fostering sustainable and health-promoting communities (17). Second, the development of spatially explicit indicator systems could provide actionable benchmarks for healthy city construction, particularly in small and medium-sized cities where data-driven planning remains underutilized (18). Despite these advances, challenges persist in embedding public health priorities within urban planning frameworks, especially in rapidly urbanizing regions. To address this, scholars advocate for comprehensive planning mechanisms that reconcile market-driven development with health equity objectives (8). Furthermore, emerging research highlights the necessity of spatially differentiated intervention strategies, urging policymakers to tailor approaches based on regional socioeconomic contexts and population heterogeneity (19, 20).

Taken together, scientists have been researching the sustainable development of healthy cities and the impacts of urban planning on public health, and have produced rich results. However, little attention has been paid in the literature to the impact of healthy urban physical activity on the public health of residents. In view of this, this study selects 84 cities in China from 2011 to 2023 as samples, and empirically analyses the influence of healthy urban movement on the public health level in China by constructing a double difference model. The marginal contributions of this study are threefold. First, by focusing on the NHCC as the research subject, we construct a Difference-in-Differences (DID) model to empirically examine the impact of healthy city initiatives on public health outcomes (21). Second, diverging from prior literature, this study rigorously addresses potential endogeneity and heterogeneity issues in evaluating the NHC’s impacts. Third, to elucidate the underlying mechanisms of policy impacts and investigate how wastewater treatment rates modulate the magnitude and direction of these impacts, we employ mediation effect tests (to identify pathways through industrial structure upgrading) and moderation effect analyses (to assess the conditional role of environmental governance).

## 3 Theoretical analysis and research hypothesis

To analyze the impact mechanism of the policy of creating a National Health City on public health, it is necessary to place it within the intersecting theoretical framework of environmental economics and public health. The Environmental Kuznets Curve (EKC) reveals an inverted U-shaped relationship between economic development and environmental pollution: In the initial stage of industrialization, economic growth often comes at the expense of environmental quality deterioration (22). However, when the per capita GDP exceeds a specific threshold, the upgrading of the industrial structure and technological progress will drive the improvement of environmental quality. This implies that by raising environmental governance standards, such as the mandatory requirement in the National Health City Standards that the sewage treatment rate should be greater than or equal to 95%, the policy of creating a National Health City can accelerate the process of cities crossing the EKC inflection point. Taking Suzhou City as an example, it has achieved a “green transition” by shutting down high-pollution textile enterprises and developing the biomedical industry. From 2015 to 2020, the industrial sulfur dioxide (SO₂) emissions decreased by 62%, and at the same time, the incidence rate of respiratory diseases significantly decreased (23, 24).

The Social Determinants of Health (SDOH) theory expands the interpretation dimension of the policy effect from a multi-dimensional perspective. The World Health Organization (WHO) has clearly pointed out that health inequalities stem from the interaction of the physical environment, the accessibility of medical services, and socio-economic status. The policy of creating a National Health City reshapes the determinants of health through three mechanisms: Firstly, the improvement of the physical environment directly reduces health risks. For example, the increase in the sewage treatment rate has led to a 23% decrease in the incidence rate of water-borne infectious diseases, as indicated by data from the National Center for Disease Control and Prevention. And the carbon emission trading scheme can significantly improve public health outcomes by incentivizing emission reductions and mitigating environmental pollution (25, 26). Secondly, the standard for the allocation of primary medical resources (establishing one community health service center for every 30,000 people) has enhanced the accessibility of medical services. According to the “Health Service Circle Coverage and Chronic Disease Management Report” of Hangzhou City’s pilot project, after the coverage rate of the 15-min health service circle increased, the compliance rate of chronic disease management increased by 18.7%. Thirdly, the green employment derived from environmental governance (for example, the environmental protection industry in Nanjing City created 12,000 new jobs in 2019) has improved the economic situation of low-income groups and alleviated the “health poverty trap.” This coordinated intervention in the environment, services, and the economy reflects the policy design logic of structural health promotion within the SDOH framework.

In the actual implementation of the NHCC, it not only focuses on directly improving the urban environment, such as strengthening the waste disposal system, increasing the capacity of sewage treatment and promoting green building standards to reduce the negative impact of environmental pollution on people’s health, but also endeavors to strengthen the construction of the primary healthcare service system, which effectively improves the accessibility and equity of healthcare services and narrows the health gap between urban and rural areas. It has also endeavored to strengthen the construction of the primary health care service system, effectively improving the accessibility and fairness of health care services and narrowing the health gap between urban and rural areas. More importantly, the health work has also fostered close cooperation between the government, communities, enterprises and residents, creating a strong synergy in social governance and improving the city’s ability to cope with public health crises (27). To ensure the quality of national health cities, China has changed the evaluation cycle from 2 to 3 years, with declarations in the first quarter of each year of the cycle, evaluations in the intervening years, and a centralized naming process in the fourth quarter of the third year. By December 2021, there were 438 national health cities (districts) in China, accounting for 57.5% of the total number of 762 cities (districts).

As a livelihood project with great brand impacts and far-reaching influence in China’s urban quality improvement strategy, the NHCC has played an irreplaceable role in promoting urban appearance innovation, strengthening public health system and promoting residents’ health and well-being in recent years. Through the formulation and implementation of a series of new standards such as city appearance and environmental sanitation improvement, disease prevention and control system construction, sewage treatment and resource utilization, the efficiency of urban sanitation environment and comprehensive health management will be improved in an all-round way (28). It not only promotes the improvement of urban infrastructure, but also significantly enhances the capacity of public health services and provides residents with a safer and more livable living environment. In view of the positive role of NHC in improving urban hygiene, preventing and controlling the spread of diseases, and improving the quality of life of residents, this study puts forward the following research hypotheses based on the existing theoretical and practical results:

*Hypothesis* 1: The establishment of a national health city will have a positive impact on public health.

Given China’s vast geographical area and complex and diverse natural environment, each region exhibits significant regional heterogeneity in terms of geographic characteristics, level of economic development, population size, and historical and cultural background. This has not only shaped their unique economic and social landscapes, but has also had a significant impact on the effectiveness of public health policies and the depth and breadth of their impact on the health of the population. In the context of the NHCC, it is imperative to undertake a thorough examination of the impact of these characteristics on the efficacy of health initiatives and their contribution to public health. This is essential for the precise implementation of policies and the optimal allocation of resources. First of all, from the perspective of natural environment and geographical location, there are significant differences in climate conditions, ecological resources and environmental carrying capacity among the northeast, eastern coastal and central and western regions of China. These differences in natural environment may lead to different implementation difficulties, cost-effectiveness and residents’ acceptance of the NHCC in different regions, and then affect their marginal contribution to public health. Therefore, this study puts forward the hypothesis:

*Hypothesis* 2: The impact of establishing a national health city on public health level exists regional heterogeneity.

The level of economic development is an important index to measure the social progress and resource input capacity of a region. Economically developed areas often have more adequate financial support and more efficient administrative management system, which may accelerate the transformation of the achievements of health creation activities into public health and well-being, and vice versa may face more challenges. Based on this, this study further assumes 3:

*Hypothesis* 3: The impact of establishing a national health city on the public health level of regions with different economic development levels is heterogeneous.

As a key factor affecting the balance between demand and supply of public health services, population size cannot be ignored in the implementation of the NHCC. Densely populated urban areas may need more refined management and services to meet the huge public health needs, while sparsely populated areas may face the problem of uneven distribution or inefficient utilization of resources. Therefore, this study puts forward:

*Hypothesis* 4: The impact of establishing a national health city on the public health level of cities with different population sizes is heterogeneous.

The optimization and upgrading of industrial structure means the transformation from high pollution and high energy consumption industries to green, low carbon, healthy and friendly industries. From the perspective of industrial relevance, new industries can promote the coordinated development of upstream and downstream; from the perspective of sustainable development, it meets the requirements of long-term development, can coordinate economic, social and environmental development, and effectively reduce environmental pollution, so research hypothesis 5 is put forward.

*Hypothesis* 5: Establishing a national health city can affect the public health level by promoting the optimization and upgrading of industrial structure.

China’s new version of National Standards for Hygienic Cities requires higher sewage treatment rate and other indicators. From the perspective of environmental policy, this urges cities to strengthen the construction and management of sewage treatment facilities. As a key factor, the level of urban sewage treatment capacity directly affects the standard of sewage treatment rate, which may further adjust the environmental quality and even public health (29). Therefore, whether and to what extent the establishment of a national health city is affected by urban sewage treatment capacity needs further empirical analysis. Therefore, research hypothesis 6 is put forward.

*Hypothesis* 6: The sewage treatment rate plays a regulatory role in the positive impact of creating a national health city on public health level.

## 4 Variables and models

### 4.1 Variable selection

#### 4.1.1 Explained variable

Public health level (impact). The level of public health is an important index to measure the basic public service level and people’s health level in countries and regions, and the improvement of public health is a core content of the evaluation standard of national excellent health cities. Due to data availability, this study uses a single negative indicator of population mortality to measure public health (30).

#### 4.1.2 Explanatory variables

The virtual variable ( $\mathrm{did}=\mathrm{trea}t_{i}\times time_{t}$ ) of the policy is the core explanatory variable of this study. Among them, $\mathrm{trea}t_{i}$ is a group virtual variable. According to the evaluation of the activities of creating a national health city, if the area is successfully selected, the value of this variable is 1, otherwise it is 0. $time_{t}$ is a virtual variable of time. According to the NHC Standard, 3 years is a review cycle. Consequently, the value of this variable is 1 if the area is in the review cycle and 0 if it is not.

#### 4.1.3 Control variables

This study selects economic development level, urbanization level, population density and medical service level as control variables. Among them, the level of economic development (PGDP) is expressed by per capita GDP. The level of urbanization (URBAN) is expressed by the ratio of the urban population in that year to the total population at the end of the year. The population density (POP) is expressed by the ratio of registered population to urban construction land area. And the level of medical service (PDOC) is expressed by the total number of medical and health institutions.

#### 4.1.4 Intermediate variables

Industrial structure (IND) reflects the proportional relationship between different industries in this region in a certain period, which is expressed by the ratio of the added value of the secondary industry to the city’s GDP in that year.

#### 4.1.5 Adjusting variables

The sewage treatment rate (WSCL) reflects the concentration of sewage in a region, the matching degree of disposal facilities and the compensation degree to the environment and health, which is of great significance for promoting sustainable development and increasing social welfare.

### 4.2 Data sources

Based on the balanced panel data of 84 cities in China from 2011 to 2023, this study examines the impact of the national health city policy on public health in China. The dependent variable, public health outcomes, measured by population mortality rates, was sourced from the China City Statistical Yearbook for respective years. Original data for indicators related to control variables, mediating variables, and moderating variables were obtained from the China Economic Information Network (CEIN) Statistical Database and the EPS Data Platform, supplemented by additional municipal health records. Missing values were addressed using the multiple imputation method to ensure dataset completeness.

### 4.3 Model selection

Based on the urban panel data from 2011 to 2023, this study studies the influence of the policy of establishing a national health city on the public health level of China, and sets the following regression model:

(1) $$\mathrm{effec}t_{\mathrm{it}}=\alpha +\mathrm{\beta trea}t_{i}\times time_{t}+\delta X_{it}+\mu_{i}+\lambda_{t}+\varepsilon_{\mathrm{it}}$$

Where i and t represent city and year respectively; the explanatory variable $\mathrm{effec}t_{\mathrm{it}}$ represents the impact of the NHCC, i.e., the level of public health; $\mathrm{trea}t_{i}$ is a dummy variable for the city in the pilot area, $\mathrm{trea}t_{i}=1$ represents the treatment group that implements the policy, $\mathrm{trea}t_{i}=0$ represents the control group; $time_{t}$ is a dummy variable for the time of the pilot policy, $time_{t}=1$ indicates the period of the NHCC impact investigation, and $time_{t}=0$ denotes the basic period of control of the NHCC; $X_{\mathrm{it}}$ is a control variable; $\mu_{i}$ and $\lambda_{t}$ are city fixed impacts and year fixed impacts, respectively; and $\varepsilon_{\mathrm{it}}$ is a random perturbation term.

### 4.4 Research design

**Figure 2:**
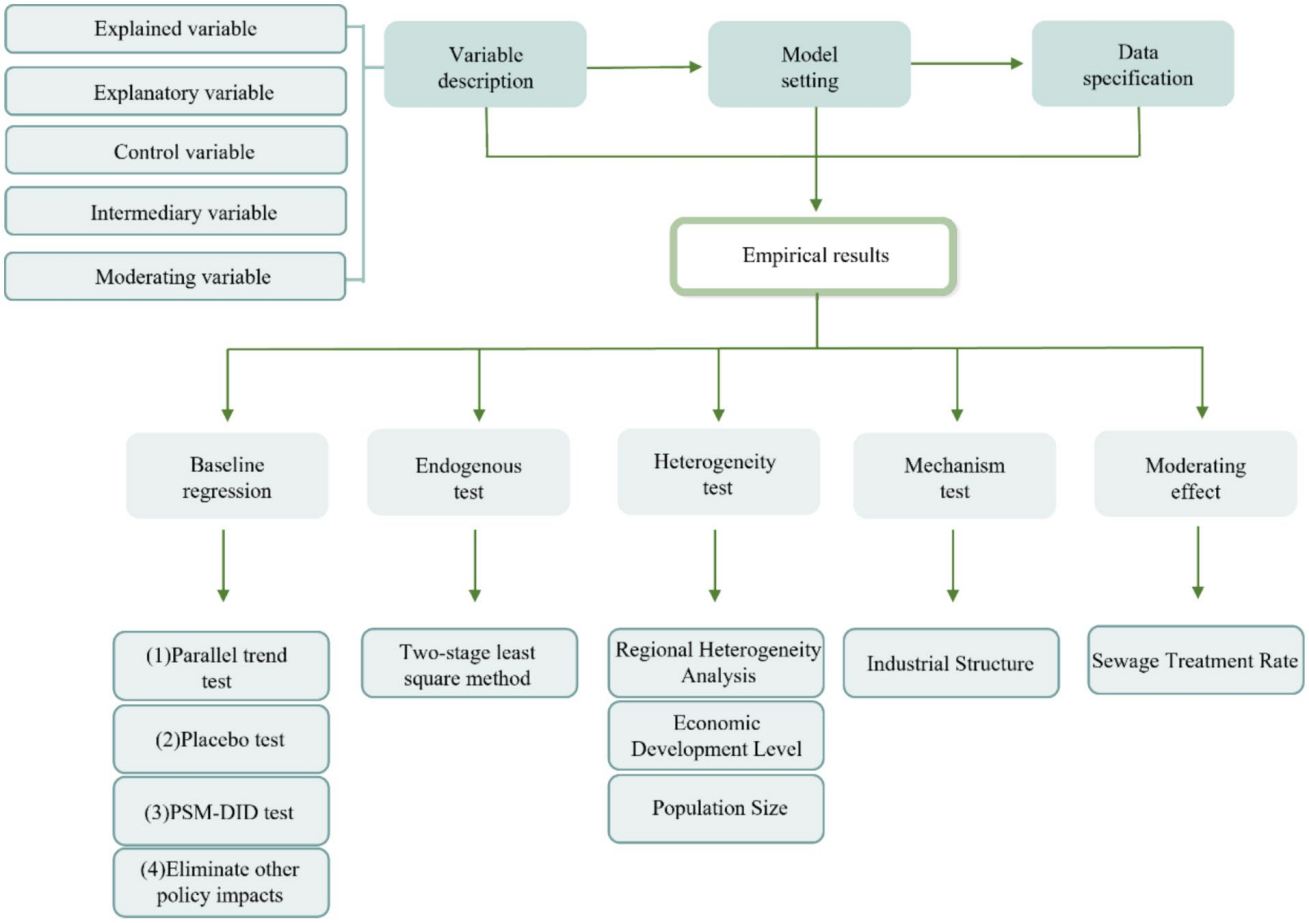
Flow chart of the research method.

The specific research design process is shown in Figure 2.

## 5 Results of empirical study

### 5.1 Descriptive statistics

Descriptive statistics of main variables are shown in Table 1.

**Table 1:** Descriptive statistics.

Variables	Obs	Mean	SD	Min	Median	Max
effect	1,092	6.834	2.033	2.780	6.500	16.590
did	1,092	0.223	0.416	0	0	1.000
lnpgdp	1,092	10.643	0.528	9.260	10.670	11.710
urban	1,092	1.025	0.461	0.450	0.970	3.860
lnpop	1,092	5.717	0.883	1.760	5.880	7.090
lnpdoc	1,092	7.545	0.885	5.470	7.580	9.840

### 5.2 Results of DID

This study mainly investigates the influence of the NHCC on the public health level of China from a macro perspective through the urban panel data from 2011 to 2023. In order to reduce the possible impact of other related variables, this study further controls the fixed impact of year and fixed impact of region on the basis of controlling the controlled variables, and regresses the benchmark regression model (1) (Equation 1). Table 2 reports the benchmark regression results of the impact of the NHCC on the public health level of residents. The results show that column (1) only controls the fixed impact of city and year, and the interaction coefficient is −0.598, which passes the significance test of 5%; in column (2), the control variable is added, and the estimation coefficient of the core explanatory variable did is significantly negative at the level of 1%. The regression results all show that the NHCC has reduced the population mortality rate to a certain extent and promoted the improvement of public health level.

**Table 2:** Benchmark regression results I.

Variables	effect	
	(1)	(2)
did	−0.598^**^	−0.676^***^
	(0.247)	(0.251)
Control	NO	YES
Year FE	YES	YES
Prov FE	YES	YES
Cons	6.967^***^	1.528
	(0.077)	(4.564)
N	1,092	1,092
R^2^	0.314	0.317

Standard errors in parents *p < 0.1, **p < 0.05, **p < 0.01. The values in parentheses are robust standard errors for clustering to the city level.

### 5.3 Robustness tests

To assess the robustness of the aforementioned regression results, this study conducts comprehensive robustness checks from four dimensions: parallel trend tests, placebo tests, PSM-DID analyses, and exclusion of confounding policy interventions. These tests collectively ensure the reliability of the baseline model estimation.

#### 5.3.1 Parallel trend test

**Figure 3:**
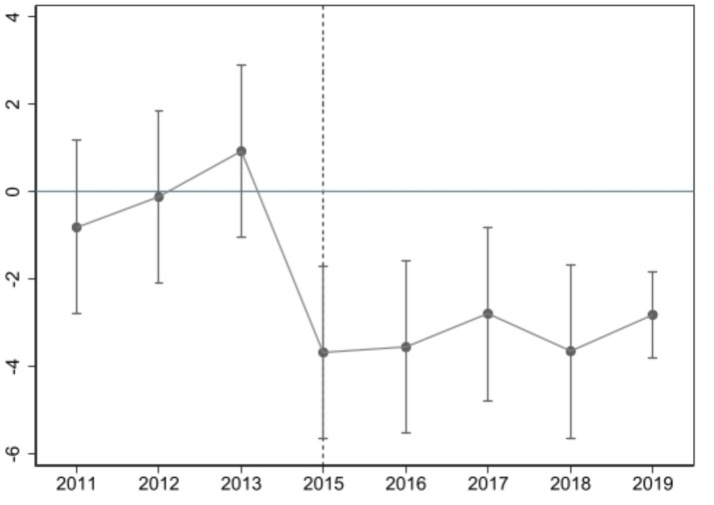
Parallel trend test.

The use of DID model requires that the experimental group and the control group have the same development trend before the policy occurs, that is, parallel trend hypothesis (31). The proposed event research method is used to test the parallel trend. In order to reduce the influence of multicollinearity, the year of national health city activities is taken as the base period, and this period is omitted in the regression. Figure 3 shows the results of parallel trend test.

It is evident that the regression coefficients are not significant prior to the centralized naming of “Healthy City,” signifying that there is no substantial difference between the experimental group and the control group with respect to the level of public health. Subsequent to the official naming of the city as a national health city, the regression coefficients exhibit a significant negative trend, indicating that the NHCC can substantially reduce the population mortality rate and enhance the level of public health. This finding is consistent with the parallel trend test. Following the official designation of the city as a national health city, the regression coefficient is found to be significantly negative, thereby indicating that the NHCC can significantly reduce the mortality rate and enhance the public health level of the residents, a finding that is in alignment with the parallel trend test. In addition, the policy impact is most pronounced in the first year after the impact of the NHCC, after which the estimated coefficient fluctuates. This may be due to the lack of a permanent and effective mechanism to avoid the “return” phenomenon. Hypothesis 1 was initially tested.

#### 5.3.2 Placebo test

**Figure 4:**
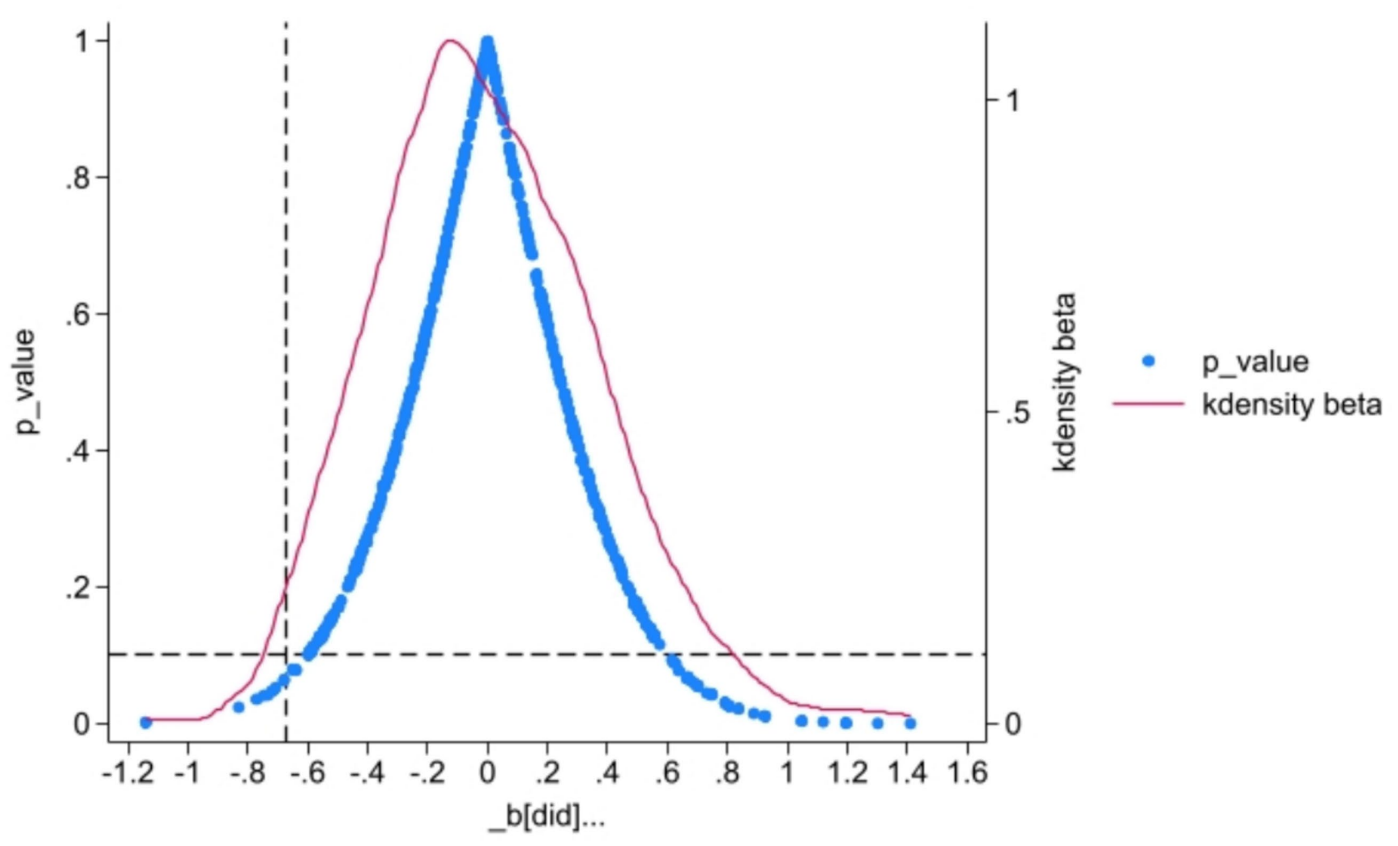
Placebo test.

In order to verify that the improvement of public health level by the NHCC is not caused by other policies in the same period or influenced by other omitted variables, this study conducts a placebo test on the basis of passing the parallel trend test to further verify. Repeat random sampling for 500 times, and perform regression according to Equation 1. Figure 4 shows that the scatter distribution of the probability density distribution of co-estimation is all located near 0, and the true coefficient (−0.676) estimated by regression based on the vertical line is much larger than the estimated value obtained by random simulation, and the coefficients represented by most scatter points are not significant at least at the level of 10%. To sum up, the positive impact of the NHCC on public health level has not been affected by other policies or other omitted variables in the same period, which further proves that regression results are robust.

#### 5.3.3 PSM-DID test

**Figure 5:**
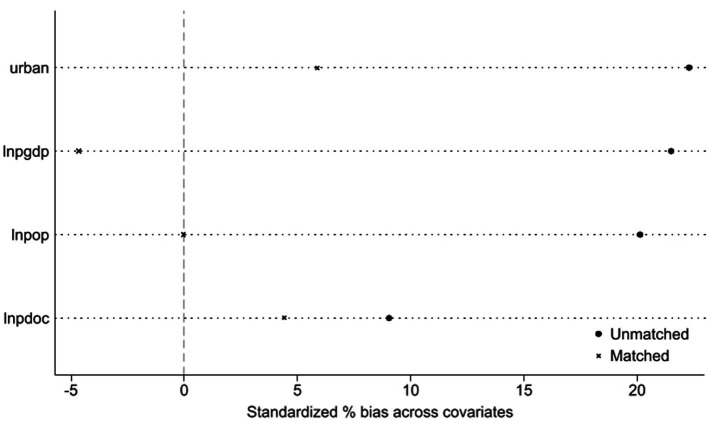
Balance test.

**Figure 6:**
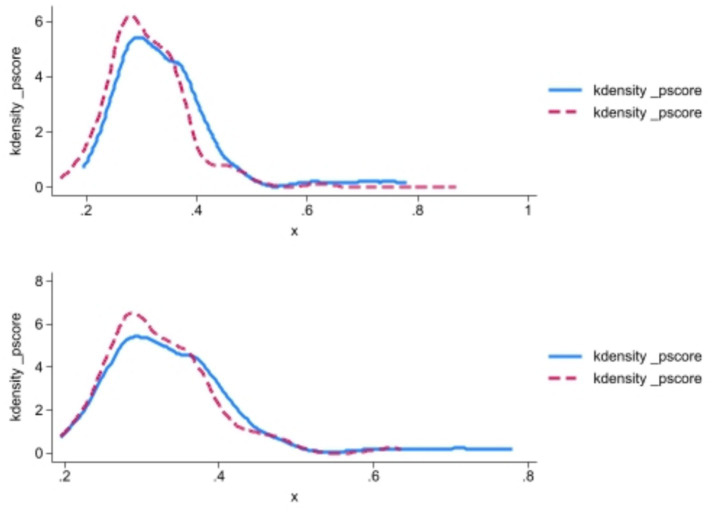
Results of matching the probability distribution density function of the score values of the forward and backward tendencies.

Because the sample selection of the NHCC is not random, there may be a problem of sample selection deviation. This study uses PSM-DID method to test the robustness. According to the evaluation results of the cycle, this study divides the panel data of 84 cities into successful cities and unsuccessful cities, and matches the tendency scores of the two groups of data. Specifically, Logit model is used to estimate the tendency score, and the nearest neighbor matching with a 1:2 matching ratio. Finally, DID regression is conducted again based on the tendency score matching. Figure 5 is the balance test result. After matching, the absolute values of standard deviation of economic development level (PGDP), urbanization level (URBAN), population density (POP) and medical service level (PDOC) all decreased greatly, indicating that there is no significant difference in the observable variables between the experimental group and the control group after matching. To further validate the matching quality, probability density function plots of propensity scores for the treatment and control groups were examined. Figure 6 demonstrates that the treatment and control groups exhibit parallel trends in propensity score distributions after matching, confirming the effectiveness of the caliper nearest neighbor matching approach (caliper width = 0.01).

Table 3 reports the regression results after matching. The estimated coefficients, symbols and significance after matching are basically consistent with the benchmark regression results, and the estimated coefficients of the interaction item (did) are all significantly negative at the level of 5%, indicating that the NHCC has significantly improved the public health level, and the model has strong robustness. Hypothesis 1 is further verified.

**Table 3:** Benchmark regression results II.

Variables	effect	
	(1)	(2)
did	−1.670^**^	−1.460^**^
	(0.679)	(0.671)
Control	YES	YES
Year FE	YES	YES
Prov FE	YES	YES
Cons	−17.424	7.430^***^
	(12.231)	(0.208)
N	1,092	1,092
R^2^	0.222	0.218

Standard errors in parents *p < 0.1, **p < 0.05, **p < 0.01. The values in parentheses are robust standard errors for clustering to the city level.

#### 5.3.4 Eliminate other policy impacts

The Air Pollution Prevention and Control Action Plan (APPCAP) implemented in 2013 prioritized regions including Beijing, Tianjin, Hebei Province, Jiangsu Province, Anhui Province, Shanghai, Zhejiang Province, and Guangdong Province. Consequently, this study excluded Tianjin; cities within Hebei Province (Tangshan, Handan, Xingtai, Baoding, Chengde, Cangzhou, and Hengshui); Lianyungang City in Jiangsu Province; cities in Anhui Province (Hefei, Wuhu, Huainan, Huaibei, Anqing, Chuzhou, Fuyang, Bozhou, Chizhou, and Xuancheng); and Yangjiang and Chaozhou in Guangdong Province. A revised regression analysis was subsequently conducted to account for these exclusions. The results in column (1) of Table 4 show that the coefficient of interaction term is still significantly negative, indicating that the APPCAP has not affected the previous empirical conclusions.

**Table 4:** Benchmark regression results III.

Variables	effect	
	(1)	(2)
did	−0.379^*^	−0.886^**^
	(0.191)	(0.403)
Control	YES	YES
Year FE	YES	YES
Prov FE	YES	YES
Cons	−0.230	−2.571
	(7.092)	(5.216)
N	739	787
R^2^	0.421	0.403

Standard errors in parents *p < 0.1, **p < 0.05, **p < 0.01. The values in parentheses are robust standard errors for clustering to the city level.

The Carbon Emission Trading Pilot (CETP) program launched in 2013 included Beijing, Tianjin, Shanghai, Chongqing, Hubei Province, Guangdong Province, and Fujian Province. To isolate the impact of the carbon trading pilot on the “National Sanitary City” campaign impacts, this study excluded Tianjin; Chongqing; cities within Hubei Province (Huangshi, Ezhou, Jingmen, Xiaogan, Jingzhou, Huanggang, and Suizhou); Yangjiang and Chaozhou in Guangdong Province; and cities in Fujian Province (Putian, Nanping, Longyan, and Ningde). A revised regression analysis was subsequently conducted to account for these exclusions. The cross-item coefficient reported in column (2) of Table 4 is still significantly negative, indicating that the CETP has little interference with the previous empirical conclusions.

### 5.4 Endogeneity test

There may be a causal relationship between the NHCC and the level of public health, that is, due to the improvement of public health level, cities are more in line with the requirements of disease prevention and control in the National Standards for Hygienic Cities, which promotes the successful selection of cities. In order to reduce the influence of endogenous factors on the regression results as much as possible, this study selects the first phase of the NHCC as the instrumental variable (iv) and adopts the two-stage least square method for regression. Table 5 reports the regression results, and column (1) shows the first-stage regression results. The statistical value of Kleibergen-Paap rk Wald *F* (131.423) is far greater than the critical value of Stock-Yogo weak instrumental variable test at 10% bias level, and there is no weak instrumental variable problem. Column (2) shows the results of the second-stage regression, and the interaction items are significantly negatively correlated at least at the level of 10%. This shows that the choice of instrumental variables is reasonable. The influence of the NHCC on public health level is basically consistent with the benchmark regression model.

**Table 5:** Benchmark regression results IV.

Variables	First stage	Second stage
	did	effect
iv	0.840^***^	
	(0.032)	
did		−1.062^*^
		(0.626)
Control	YES	YES
Year FE	YES	YES
Prov FE	YES	YES
Cons	−1.689^***^	0.575
	(0.214)	(3.695)
N	1,092	1,092
R^2^	0.423	0.004

Standard errors in parents *p < 0.1, **p < 0.05, **p < 0.01. The values in parentheses are robust standard errors for clustering to the city level.

## 6 Further analysis

### 6.1 Heterogeneity test

#### 6.1.1 Regional heterogeneity analysis

In consideration of China’s substantial geographical expanse and the potential for significant variations across diverse regions, this study has been undertaken to divide the sample into three distinct categories: the northeastern region, the eastern coastal region, and the central and western regions. The northeastern region includes Tongliao City, Yingkou City, Liaoyang City, Panjin City, Liaoyuan City, Harbin City. The eastern coastal region comprises: Tianjin City, Tangshan City, Handan City, Xingtai City, Baoding City, Chengde City, Cangzhou City, Hengshui City, Lianyungagn City, Putian City, Nanping City, Longyan City, Ningde City, Jining City, Heze City, Yangjiang City, Chaozhou City, and Haikou City. The central and western regions: Taiyuan City, Shuozhou City, Jinzhong City, Hefei, Wuhu, Huainan, Huaibei, Pingxiang, Xinyu, Yingtan, Hebi, Huangshi and so on. The regression results are displayed in Table 6. Columns (1), (2) and (3) correspond to the Northeast, East Coast and Midwest regions, respectively. The regression results demonstrate that the East Coast region is significant at the 1% level of significance, whilst the Northeast and Midwest regions are not significant. This indicates that the NHCC is effective in reducing public health risks in the East Coast region, but not in the Northeast and Midwest regions. Furthermore, there is regional heterogeneity in the positive impacts of the NHCC. This finding lends further credence to Hypothesis 2, which was previously postulated and is now verified. The NHCC is shown to be effective in reducing public health risks in the eastern coastal region, but not in the northeastern and central and western regions. The positive impacts of the NHCC are found to be heterogeneous across these regions.

**Table 6:** Benchmark regression results V.

Variables	effect		
	Northeast	East	Midwest
did	8.210	−9.706^***^	−0.440
	(7.103)	(1.937)	(0.277)
Control	YES	YES	YES
Year FE	YES	YES	YES
Prov FE	YES	YES	YES
Cons	−166.580	86.596^**^	3.541
	(165.633)	(33.515)	(5.517)
N	78	234	780
R^2^	0.427	0.394	0.311

Standard errors in parents *p < 0.1, **p < 0.05, **p < 0.01. The values in parentheses are robust standard errors for clustering to the city level.

The possible reasons analyzed in this article are as follows: Firstly, there are differences in the level of infrastructure construction. Public resources (such as the number of medical and health institutions, the number of health personnel, etc.) in the eastern coastal areas are richer than those in the northeastern region and the central and western regions. In eastern coastal cities, due to the relative advantages in economic development and resource allocation, environmental governance facilities are relatively complete, enabling a rapid improvement in public health levels (32). Secondly, there are differences in the industrial structure. The northeastern region and the central and western regions mainly focus on heavy industries, while the eastern coastal areas mainly focus on manufacturing and light industries. This transformation and upgrading of the industrial structure not only promote economic growth but also improves environmental sanitation and the health status of residents. In addition, due to the more stringent implementation of environmental protection regulations, the withdrawal or transformation of heavily polluting enterprises has led to an overall improvement in the environmental quality of the eastern coastal areas. However, most of the northeastern, central and western regions rely on heavy industries and resource-based industries, facing more serious environmental pollution problems. The implementation of the NHCC policy in these regions encounters more challenges, affecting the effectiveness of policy implementation. Thirdly, there are differences in residents’ health awareness. In eastern coastal cities, due to the relatively high per capita income, residents have a stronger health awareness and are more willing and active in supporting health policies. Therefore, a series of measures of the NHCC policy are more easily accepted by residents, thus forming a virtuous cycle. In the central and western regions and the northeastern region, the relatively low income levels and the degree of health education of residents may limit their support for and compliance with public health policies. These reasons may lead to a more obvious improvement in the public health level of residents in the eastern coastal areas.

#### 6.1.2 Heterogeneity analysis by economic development level

Due to the differences in the economic development level of different regions in China, the fiscal expenditure of local governments on health and the amount of environmental pollution control may lead to differences in the public health level of different cities. Therefore, this study takes the average per capita GDP of the sample as the standard, and divides the sample into high economic development level and low economic development level for regression. The regression results are shown in Table 7. The first column is a region with high economic development level, and the second column is a region with low economic development level. It can be seen that the region with high economic development level is significant at the level of 10%, while the region with low economic development level is not significant.

**Table 7:** Benchmark regression results VI.

Variables	effect	
	High economic level	Low economic level
did	−0.236^*^	−0.250
	(0.135)	(0.175)
Control	YES	YES
Year FE	YES	YES
Prov FE	YES	YES
Cons	−6.226^**^	−3.119
	(2.539)	(2.181)
N	472	420
R^2^	0.160	0.098

Standard errors in parents *p < 0.1, **p < 0.05, **p < 0.01. The values in parentheses are robust standard errors for clustering to the city level.

This study holds that this may be because public health services and medical resources are superior in areas with high economic development level, while areas with low economic development level not only lack public health services and medical resources, but also may be deeply affected by environmental problems brought about by economic development. Relatively developed regions can usually provide sufficient financial resources to support the improvement of medical facilities and infrastructure. In contrast, economically backward regions may experience a lag in the construction of public health infrastructure due to limited resources. In regions with high economic development, such as the eastern coastal cities, the government has substantial financial strength and is capable of making more investments in public health, thereby enhancing the effectiveness of the NHCC. On the contrary, regions with a relatively weak economy often face the problem of insufficient medical and health resources, which affects the effective implementation of the policy. To sum up, this study found that the impact of the NHCC on public health level is heterogeneous in economic development level. Hypothesis 3 is verified.

#### 6.1.3 Heterogeneity analysis by population size

In order to investigate whether the positive impact of the NHCC exists the heterogeneity of population size, this study divides the sample into high population size and low population size for regression based on the average of the total sample population. The regression results are shown in Table 8. The first column is high-population area, and the second column is low-population area. It can be seen that the policy impact of high-population area is not significant, and the policy impact of low-population area is significant at 1% level. That is to say, the NHCC has a significant effect on alleviating public health risks in low-population areas. This may be because areas with low population scale have more abundant medical and health resources, natural resources and material resources, and the traffic and housing conditions are more impressive, which is conducive to creating a good living environment and improving public health. In addition, in regions with a low population size, the coordination between infrastructure and public services is better. The community cohesion and the willingness to cooperate are relatively strong, enabling effective cooperation in the implementation of government policies and forming a sound social support network. In contrast, in regions with a high population size, due to the complex demands and scattered individuals, the collaboration between communities may not be as smooth as that in regions with a low population size, further weakening the enhancing effect of public health policies. Therefore, the impact of the NHCC on public health level is heterogeneous in population size. Hypothesis 4 is verified.

**Table 8:** Benchmark regression results VII.

Variables	effect	
	High population size	Low population size
did	0.002	−0.901^***^
	(0.480)	(0.295)
Control	YES	YES
Year FE	YES	YES
Prov FE	YES	YES
Cons	0.274	−0.297
	(9.307)	(5.750)
N	269	822
R^2^	0.294	0.343

Standard errors in parents *p < 0.1, **p < 0.05, **p < 0.01. The values in parentheses are robust standard errors for clustering to the city level.

### 6.2 Mediation effect test

According to the above analysis, the NHCC may affect the level of public health through industrial structure. To verify this conjecture, this study draws lessons from Wen Zhonglin and Ye Baojuan (33). The proposed mediation analysis model takes industrial structure as a mediating variable for regression. Table 9 reports the test results of the intermediary impact of the NHCC on public health level. The first column regresses the NHCC with the public health level as the explained variable, and the results show that the policy can significantly improve the public health level. The second column regresses the NHCC with the industrial structure as the explained variable, and the results show that it has a significant role in promoting the industrial structure. Column (3) verifies the mediation effect of industrial structure in the process of the NHCC to improve public health level. The industrial structure is significant at the level of 5%, so it can be judged that the mediation effect of industrial structure is significant. The NHCC requires cities to improve environmental sanitation and quality of life, urge cities to close or relocate heavily polluted industrial enterprises, and encourage the development of clean and environmentally friendly industries. At the same time, reducing the proportion of heavy industry and manufacturing industry may reduce air pollution and water pollution, thus reducing the incidence of respiratory diseases and water-borne diseases. The development of service industry and high-tech industry may attract more talents with high skills and high income, and these people often have higher health awareness and better health habits. Hypothesis 5 is verified.

**Table 9:** Benchmark regression results VIII.

Variables	(1)	(2)	(3)
	effect	lnind	effect
did	−0.507^**^	0.117^*^	−0.513^**^
	(0.239)	(0.068)	(0.240)
Control	YES	YES	YES
Year FE	YES	YES	YES
Prov FE	YES	YES	YES
Cons	0.611	4.505^***^	0.377
	(3.909)	(1.677)	(3.933)
N	952	952	952
R^2^	0.386	0.790	0.386

Standard errors in parents *p < 0.1, **p < 0.05, **p < 0.01. The values in parentheses are robust standard errors for clustering to the city level.

### 6.3 Moderation analysis

Examining the moderating effect of the rate of wastewater treatment in the process of the NHCC affecting the level of public health, the regression results are shown in Table 10. Column (1) shows that the interaction coefficient is significantly negative at the level of 5%, which is consistent with the benchmark regression result; column (2) shows that the sewage treatment rate, the coefficient of the interaction term (X_D) between the sewage treatment rate and the original independent variable are all significantly negative, indicating that sewage treatment has strengthened the negative regulation of the NHCC on public health risks. Hypothesis 6 is verified.

**Table 10:** Benchmark regression results IX.

Variables	effect	
	(1)	(2)
did	−0.453^**^	−0.348
	(0.204)	(0.249)
lnwscl		−0.905^**^
		(0.328)
X_D		−3.262^***^
		(1.014)
Control	YES	YES
Year FE	YES	YES
Prov FE	YES	YES
Cons	1.725	5.666
	(3.173)	(3.822)
N	952	951
R^2^	0.406	0.409

Standard errors in parents *p < 0.1, **p < 0.05, **p < 0.01. The values in parentheses are robust standard errors for clustering to the city level.

## 7 Conclusion and recommendations

### 7.1 Research conclusion

This study conducts a series of empirical studies to investigate the public health improvement impacts of the NHCC. First, this study takes the NHCC as a quasi-natural experiment and applies the DID model and the PSM-DID model to examine the impact of the NHCC on China’s public health level. Second, to ensure the robustness of the regression results, this study conducts the parallel trend test, placebo test, PSM-DID test, exclusion of other policy impacts and endogeneity test to exclude the interference of other policies and unobserved variables. Finally, in order to further discuss whether there is heterogeneity in the positive impacts of the NHCC on public health, and to find the ways in which the NHCC affects public health and other omitted variables, this chapter conducts heterogeneity tests, mediation effect tests, and moderation effect analyses.

The results showed that the benchmark regression passed the significance test of 1%, indicating that the NHCC reduced the population mortality rate to a certain extent and promoted the improvement of public health level; the results of parallel trend test, placebo test, PSM-DID test, excluding other policy impacts and endogeneity test all show that the impact of the NHCC has significantly improved the level of public health, and the model has strong robustness. In terms of heterogeneity, the NHCC can effectively reduce the public health risk in the eastern coastal areas, but it has no significant impact on the promotion of the northeast and central and western regions, and the positive impact of the NHCC has regional heterogeneity; the impact of the NHCC is significant in areas with high economic development level, but not in areas with low economic development level, and the positive impact of the policy has heterogeneity in economic development level. The mitigation effect of the NHCC on public health risks in low-population areas is significantly higher than that in high-population areas, and the positive impact of the policy is heterogeneous in population size. In addition, this study also found that the establishment of a national health city can positively improve the public health level by promoting the optimization and upgrading of industrial structure. The sewage treatment rate plays a significant regulatory role in the positive impact of the NHCC on public health level.

### 7.2 Policy recommendations

First, the study found that the effects of the NHCC were initially strong and then weakened, with the strongest effects in the early stages of policy implementation and weakening thereafter. The reason for this may be the lack of a systematic and long-term effective mechanism to avoid the “return” phenomenon. To ensure the sustainability of the policy impacts of the NHCC, it is recommended to establish a long-term mechanism featuring an operational, supportive, supervisory and accountable mechanism, with the government playing a leading role and multiple parties collaborating (34). At the operation level, a special management office should be established under the leadership of the municipal health department. The office should regularly convene cross-departmental joint meetings to promote tasks such as garbage classification and sewage treatment, and incorporate the work achievements into the government performance assessment. In terms of support, it is necessary to strengthen the coordination between finance and technology. The central government’s finance department can set up a special fund to match local investments, with a focus on supporting the construction of sewage treatment facilities and community medical services (35, 36). Private enterprises should be encouraged to participate in the development of intelligent environmental sanitation technologies through the Public-Private Partnership (PPP) model, and tax incentives should be provided for them. At the supervision level, the system of grid supervisors in communities should be implemented. Relying on a digital platform, environmental problems should be reported in real time, and it is required that the disposal rate of these problems within 48 h should reach more than 90%. Regarding accountability, a red and yellow card warning mechanism should be established. For cities that fail to make effective rectifications, environmental protection approvals should be suspended, and enterprises that shirk their responsibilities should be included in the blacklist for bidding. By clarifying the division of labor and quantifying the objectives, a governance closed loop with multi-party linkage can be formed to ensure the long-term implementation of the policies.

Second, policy implementation should be tailored to different regions, levels of economic development and population sizes (37). Regarding regional coordinated development, a three-chain coordination mechanism for the gradient transfer of experiences from the eastern region to the central and western regions ought to be established. Through the establishment of a regional health city alliance and the construction of an environmental governance technology transfer platform, the well-developed experiences of eastern cities in areas like sewage treatment technology and garbage resource utilization can be modularly encapsulated, thus forming a replicable and promotable technical standard package. Given the characteristics of heavy-industry agglomeration in the central and western regions, a differentiated industrial exit compensation mechanism should be devised. For instance, special transformation funds could be established to support the technological upgrading of traditional enterprises. Meanwhile, as a supplementary measure, the “Pairing Plan of Medical Resources between the East and the West” should be carried out. By means of specific approaches such as building branch hospitals of Class III Grade A hospitals and deploying remote diagnosis and treatment systems, the cross-regional allocation of high-quality medical resources can be achieved. When it comes to coordinating industrial transformation and the allocation of health resources, a dual-indicator dynamic monitoring system needs to be set up. Set quantified targets, such as reducing the proportion of the secondary industry by no less than 1.2 percentage points on an annual average and increasing the number of beds in medical and health institutions per thousand people by 2.5% on an annual average. Additionally, a correlation model between industrial pollution emissions and changes in the regional disease spectrum should be constructed, and health indicators like the incidence rate of respiratory diseases should be incorporated into the access evaluation system of industrial parks.

Third, based on the results of mediation effect and moderation effect, on the one hand, in the aspect of industrial structure optimization and upgrading, local governments should increase policy support, formulate and implement a series of preferential policies, such as financial subsidies, tax relief, financial support, etc., to encourage enterprises to increase investment in research and development and promote technological innovation and industrial upgrading; guide the coordinated development of industries, promote the integrated development of different industries, and form a synergistic impact between the upstream and downstream of the industrial chain, such as promoting the combination of medical industry and information technology industry and developing smart medical care; promote the integration of health service industry and tourism industry and develop health tourism industry. On the other hand, based on the results of the moderating effect test, it is recommended to set the sewage treatment rate of 92% as the inflection point of the policy effect. For cities that fail to meet the standard, a tiered fiscal subsidy policy should be implemented. When the treatment rate is within the range of 85 to 92%, the central finance will provide a special subsidy of 5 million yuan per year for every 1 percentage point increase. Once it exceeds 92%, an incentive mechanism for maintaining the achieved results will be adopted. Meanwhile, a dynamic response model between the effluent quality of sewage treatment plants and the incidence rate of regional infectious diseases should be established, and health risk weights should be assigned to key indicators such as COD and ammonia nitrogen for management. Furthermore, mandatory clauses of Health Impact Assessment need to be embedded in the policy documents. It is required that community health service centers should be constructed simultaneously during the building of newly established industrial parks, and ensure that the spatial matching degree between the service radius of these centers and the residential areas of industrial workers reaches over 90%, so as to generate a spatial coupling effect between environmental governance and health services (38).

## 8 Limitations and future prospects

This study is not without several limitations attributable to data availability and methodological limitations. (1) Based on the availability of data, this study uses only the population mortality rate to measure the level of public health, and does not include indicators such as life expectancy, infectious disease mortality rate and chronic disease incidence rate in the indicator system, and subsequent research can consider constructing a public health indicator system. (2) Although this study has found that the sewage treatment rate has a significant mediating effect in the improvement of public health, it should be noted that there are certain limitations in using the sewage treatment rate as a single proxy indicator for environmental quality. For example, the environmental quality of a city is also influenced by multi-dimensional indicators such as air quality (e.g., PM2.5 concentration), the solid waste treatment rate, and the level of soil pollution control (39, 40). Future research could enhance the comprehensiveness of the evaluation by integrating the composite environmental index specified in the “Detailed Rules for the Implementation of the Performance Assessment of the Battle against Pollution” issued by the Ministry of Ecology and Environment.

## Data Availability

The original contributions presented in the study are included in the article/supplementary material, further inquiries can be directed to the corresponding authors.
